# ﻿A new species of *Cyrtodactylus* (Squamata, Gekkonidae) from Hon Tre Island in Khanh Hoa Province, Vietnam

**DOI:** 10.3897/zookeys.1253.149459

**Published:** 2025-09-24

**Authors:** Quyen Hanh Do, Hanh Thi Ngo, Truong Quang Nguyen, Minh Duc Le, Thomas Ziegler, Dang Trong Do, Cuong The Pham

**Affiliations:** 1 Institute of Zoology, University of Cologne, Zülpicher Str. 47b, D-50674 Cologne, Germany Institute of Biology, Vietnam Academy of Science and Technology Hanoi Vietnam; 2 AG Zoologischer Garten Köln, Riehler Str. 173, D-50735 Cologne, Germany University of Cologne Cologne Germany; 3 Institute of Biology, Vietnam Academy of Science and Technology, 18 Hoang Quoc Viet Road, Hanoi, Vietnam AG Zoologischer Garten Köln Cologne Germany; 4 Central Institute for Natural Resources and Environmental Studies, Vietnam National University, Hanoi, 19 Le Thanh Tong, Hanoi, Vietnam Vietnam National University Hanoi Vietnam; 5 Graduate University of Science and Technology, Vietnam Academy of Science and Technology, 18 Hoang Quoc Viet Road, Hanoi, Vietnam Graduate University of Science and Technology Hanoi Vietnam; 6 Faculty of Environmental Science, VNU University of Science, 334 Nguyen Trai Road, Hanoi, Vietnam VNU University of Science Hanoi Vietnam; 7 Department of Herpetology, American Museum of Natural History, Central Park West at 79th Street, New York, New York 10024, USA Department of Herpetology, American Museum of Natural History New York United States of America; 8 Faculty of Natural Sciences, Phu Yen University, Tuy Hoa Ward, Dak Lak Province, Vietnam Phu Yen University Tuy Hoa Ward Vietnam

**Keywords:** *Cyrtodactylus arnei* sp. nov., *Cyrtodactylus irregularis* group, morphology, phylogenetic relationships, taxonomy

## Abstract

A new species of the genus *Cyrtodactylus* is described from Khanh Hoa Province, South-central Vietnam based on genetic divergence and morphological differences. *Cyrtodactylus arnei***sp. nov.** is distinguished from the remaining Indochinese bent–toed geckos of the *Cyrtodactylus irregularis* group by having the unique combination of the following characteristics: size medium (SVL 70.9–78.0 mm); dorsal tubercles in 15–17 irregular rows; 34 or 35 ventral scale rows; 12–15 enlarged femoral scales on each side, in continuous series without gap between precloacal and femoral scales; precloacal pores absent in females, 5–7 in males, in a continuous row; femoral pores absent; postcloacal spurs 0–3 on each side; 19–21 lamellae under toe IV; dorsal pattern between limb insertions consisting of four narrow light bands with dark edges and a transversal row of dark spots in the middle; subcaudal scales enlarged, forming broad transverse plates. In phylogenetic analyses, the new species was nested within the *Cyrtodactylus irregularis* group without any clear sister taxon. Genetically, *Cyrtodactylus arnei***sp. nov.** is divergent from other species within the *Cyrtodactylus irregularis* group by at least 10.97% (COI) and 14.39% (ND2) based on two fragments of the mitochondrial gene.

## ﻿Introduction

South-central Vietnam is considered as a transition zone between the Central Highlands and the coastal areas below 1,000 m in elevation. Its natural habitat is characterized by evergreen forest on low-lying mountains and thorny vegetation coverage, with drought–tolerant trees such as minnows living in windy areas with little rain in coastal areas (Sterling et al. 2006). The region is recognized as a center for new reptile discoveries in Vietnam, as numerous new species have been recently described from the surrounding area, viz. *Acanthosaura murphyi* Nguyen, Do, Hoang, Nguyen, McCormack, Nguyen, Orlov, Nguyen & Nguyen, 2018; *Sphenomorphus yersini* Nguyen, Nguyen, Nguyen, Orlov & Murphy, 2018; *Cyrtodactylus phumyensis* Ostrowski, Do, Le, Ngo, Pham, Nguyen, Nguyen & Ziegler, 2020; *C. chungi* Ostrowski, Le, Ngo, Pham, Phung, Nguyen & Ziegler, 2021; *C. raglai* Nguyen, Duong, Grismer & Poyarkov, 2021; *Gekko phuyenensis* Nguyen, Nguyen, Orlov, Murphy, & Nguyen, 2021, *Cyrtodactylus orlovi* Do, Phung, Ngo, Le, Ziegler, Pham & Nguyen, 2021, *C. tayhoaensis* Do, Do, Le, Ngo, Ziegler, Nguyen & Pham, 2023, *C. arndti* Ngo, Horman, Le, Pham, Phung, Do, Ostrowski, Nguyen & Zieger, 2023; *C. binhdinhensis* Ngo, Do, Do, Pham, Bui, Ho, Nguyen, Ziegler & Le, 2024; *Lycodon anakradaya* Nguyen, Duong, Wood & Grismer, 2022; *L. truongi* Nguyen, Duong, Wood & Grismer, 2022; *Trimeresurus cyanolabris* Idiiatullina, Nguyen, Bragin, Pawangkhanant, Le, Vogel, David & Poyarkov, 2024; and *Colubroelaps adleri* Poyarkov, Bragin & Bragin, 2024 (Nguyen et al. 2018a, b, 2021a, b, 2022a; Ostrowski et al. 2020, 2021; Do et al. 2021, 2023; Ngo et al. 2023, 2024; Idiiatullina et al. 2024; Poyarkov et al. 2024).

In terms of species richness of bent–toed geckos, South-central Vietnam harbors 16 species of *Cyrtodactylus*: *C. arndti* Ngo, Horman, Le, Pham, Phung, Do, Ostrowski, Nguyen & Zieger; *C. binhdinhensis* Ngo, Do, Do, Pham, Bui, Ho, Nguyen, Ziegler & Le; *C. caovansungi* Orlov, Nguyen, Nazarov, Ananjeva & Nguyen; *C. chungi* Ostrowski, Do, Le, Ngo, Pham, Nguyen, Nguyen & Ziegler; *C. cucdongensis* Schneider, Phung, Le, Nguyen & Ziegler; *C. culaochamensis* Ngo, Grismer, Pham & Wood; *C. kingsadai* Ziegler, Phung, Le & Nguyen; *C. orlovi* Do, Phung, Ngo, Le, Ziegler, Pham & Nguyen; *C. phumyensis* Ostrowski, Le, Ngo, Pham, Phung, Nguyen & Ziegler; *C. phuocbinhensis* Nguyen, Le, Tran, Orlov, Lathrop, MacCulloch, Le, Jin, Nguyen, Nguyen, Hoang, Che, Murphy & Zhang; *C. pseudoquadrivirgatus* Rösler, Vu, Nguyen, Ngo & Ziegler; *C. raglai* Nguyen, Duong, Grismer & Poyarkov; *C. sangi* Pauwels, Nazarov, Bobrov & Poyarkov; *C. takouensis* Ngo & Bauer; *C. tayhoaensis* Do, Do, Le, Ngo, Ziegler, Nguyen & Pham; *C. yangbayensis* Ngo & Chan. All species in this area belong to the *Cyrtodactylus irregularis* group, which occurs in South-central Vietnam and extends into eastern Cambodia and southeast Laos, and includes 30 species to date (Orlov et al. 2007; Ngo and Bauer 2008; Rösler et al. 2008; Ngo and Chan 2010; Nguyen et al. 2013; Ziegler et al. 2013; Schneider et al. 2014; Pauwel et al. 2018; Ngo et al. 2020; Ostrowski et al. 2020, 2021; Do et al. 2021, 2023; Grismer et al. 2021; Nguyen et al. 2021a; Ngo et al. 2023, 2024; Uetz et al. 2025).

Recent field work on Hon Tre Island of Khanh Hoa Province, South-central Vietnam resulted in the collection of nine specimens of a gekkonid species, which can be assigned to the genus *Cyrtodactylus* based on morphological features and phylogenetic analyses. However, this *Cyrtodactylus* population showed genetic divergence and morphological differences from other known species. Thus, we herein describe a new species of *Cyrtodactylus* from Hon Tre Island of Khanh Hoa Province in South-central Vietnam.

## ﻿Materials and methods

### ﻿Sampling

**Figure 1.**
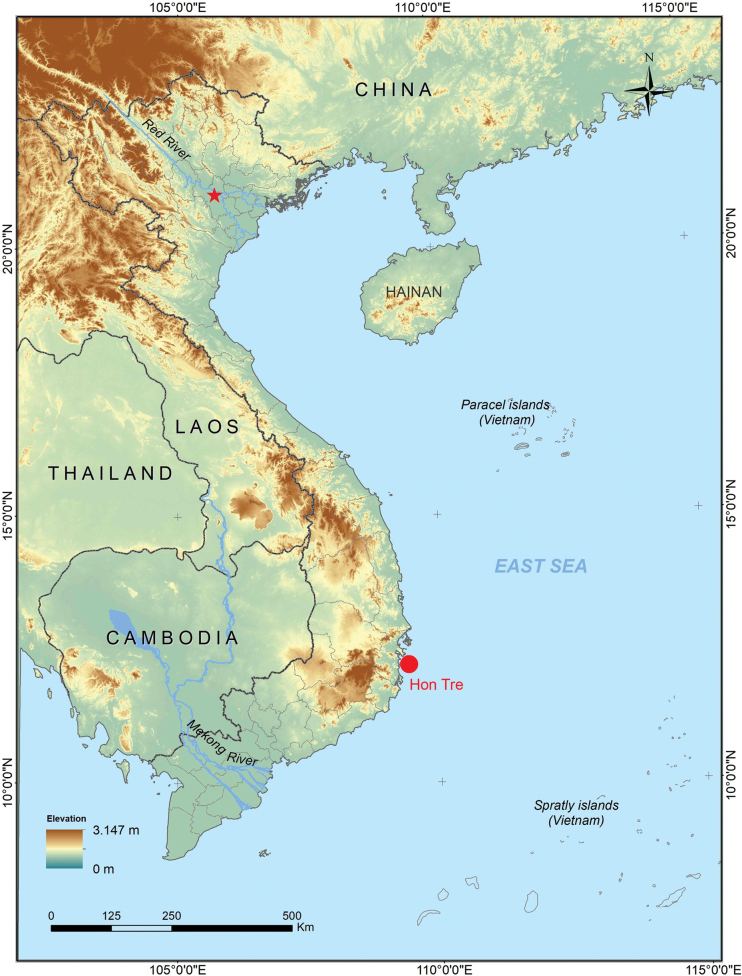
Type locality of *Cyrtodactylus arnei* sp. nov. in Hon Tre Island, Khanh Hoa Province (red dot), Vietnam (Source: NASA Shuttle Radar Topography Mission (SRTM) 2013). The capital Hanoi is indicated with a red star.

A field survey was conducted on Hon Tre Island, Khanh Hoa Province in September 2020 (Fig. 1). Specimens were anaesthetized and euthanized in a closed vessel with a piece of cotton wool containing ethyl acetate (Simmons 2002), fixed in 85% ethanol for eight hours, then later transferred to 70% ethanol for permanent storage. Specimens were deposited in the collections of the Institute of Biology (**IB**) (formerly known as the Institute of Ecology and Biological Resources **IEBR**), Hanoi, Vietnam.

### ﻿Molecular data and phylogenetic analyses

All recognized species of the *Cyrtodactylus irregularis* group were included in the study, except *C. buchardi* and *C. pseudoquadrivirgatus* sensu stricto (Suppl. material 1). Two taxa, *C. spelaeus* Nazarov, Poyarkov, Orlov, Nguyen, Milto, Martynov, Konstantinov & Chulisov, 2014 and *C. wayakonei* Nguyen, Kingsada, Rösler, Auer & Ziegler, 2010 were used as outgroups based on their phylogenetic relationships to the *C. irregularis* species group as reported by Luu et al. (2017) and Grismer et al. (2021).

DNA was extracted using DNeasy Blood and Tissue kit (Qiagen, Germany) following the manufacturer’s instructions. Extracted DNA was amplified by HotStarTaq PCR mastermix (Qiagen, Germany) with 21 µl volume (10 µl of mastermix, 5 µl of water, 2 µl of each primer at 10 pmol·ml–1 and 2 µl of DNA). PCR conditions were 95 °C for 15 minutes to active the taq; with 35 cycles at 95 °C for 30s, 45 °C for 45s, 72 °C for 60s; and a final extension at 72 °C for 6 minutes. Two fragments of the mitochondrial gene, cytochrome c oxidase subunit I (COI) and NADH dehydrogenase subunit 2 (ND2) were amplified using the primer pair VF1 d (5’–TTCTCAACCAACCACAARGAYATYGG–3’) and VR1 d (5’–TAGACTTCTGGGTGGCCRAARAAYCA–3’) (Ivanova et al. 2006) and MetF1 (5’–AAGCTTTCGGGCCCATACC–3’) and COIR1 (5’–AGRGTGCCAATGTCTTTGTGRTT–3’) (Arevalo et al. 1994; Macey et al. 1997). PCR products were visualized using electrophoresis through a 2% agarose gel stained with ethidium bromide. Successful amplifications were purified to eliminate PCR components using GeneJET^TM^ PCR Purification kit (ThermoFisher Scientific, Lithuania). Purified PCR products were sent to FirstBase (Malaysia) for sequencing in both directions.

Each gene dataset was initially aligned separately by ClustalX v. 2.1 (Thompson et al. 1997) with default settings for complete alignment. Combined data (COI + ND2) were analyzed using Bayesian inference (**BI**) as implemented in MrBayes v. 3.2.7 (Ronquist et al. 2012), Maximum likelihood (**ML**) as implemented in IQ–TREE v. 2.4.0 (Minh et al. 2020), and Maximum Parsimony (**MP**) implemented in PAUP*4.0b10 (Swofford 2001). Additionally, each gene dataset was also analyzed using BI and ML for the references. For MP analysis, a heuristic analysis was conducted with 100 random taxon addition replicates using tree–bisection and reconnection (**TBR**) branch–swapping algorithm, with no upper limit set for the maximum number of trees saved. Bootstrap support was calculated using 1,000 pseudo–replicates (BP) and 100 random taxon addition replicates. All characters were equally weighted and unordered. For the ML analysis, we employed a single model and 10,000 ultrafast bootstrap replications (**UFB**). The optimal model for nucleotide evolution was determined using jmodeltest v. 2.1.10 (Darriba et al. 2012).

For the Bayesian analyses, we used the optimal model determined by jmodeltest with parameters estimated by MrBayes v. 3.2.7. Two independent analyses with four Markov chains (one cold and three heated) were run simultaneously for 10^7^ generations with a random starting tree and sampled every 1,000 generations. Loglikelihood scores of sample points were plotted against generation time to detect stationarity of the Markov chains. Trees generated prior to stationarity were removed from the final analyses using the burn–in function. The posterior probability values (PP) for all clades in the final majority rule consensus tree were provided. Nodal support was evaluated using BP as estimated in PAUP, UFB in IQ–TREE v. 2.4.0, and PP in MrBayes v. 3.2.7. UFB > 95, and PP ≥ 95% and BP ≥ 70% were regarded as strong support for a clade (Hillis and Bull 1993; Ronquist et al. 2012; Hoang et al. 2018). The optimal model for nucleotide evolution was set to GTR+I+G for Bayesian analysis and TIM1+I+G for ML analysis. The cut–off point for the burn–in function was set to delete 25% of the total number of trees generated. Uncorrected pairwise divergences were calculated in PAUP*4.0b10.

### ﻿Morphological characteristics

Measurements were taken with a digital caliper to the nearest 0.1 mm. Abbreviations are as follows:

**SVL** snout–vent length (from tip of snout to vent);

**TaL** tail length (from vent to tip of tail, with * meaning regenerated tail);

**HL** head length (from tip of snout to retroarticular process of jaw);

**HW** head width (maximum width of head);

**HH** head height (from occiput to underside of jaws);

**OD** orbital diameter (greatest horizontal diameter of orbit);

**SE** snout to eye distance (from tip of snout to anterior–most point of eye);

**EE** eye to ear distance (from anterior edge of ear opening to posterior margin of eye);

**NE** nares to eye distance (from anterior–most point of eye to posterior–most point of nostril);

**ED** ear length (longest dimension of ear);

**ForeaL** forearm length (taken on the dorsal surface from the posterior margin of the elbow while flexed 90° to the inflection of the flexed wrist);

**CrusL** crus length (taken on the ventral surface from the posterior surface of the knee while flexed 90° to the base of heel);

**TrunkL** trunk length (from axilla to groin measured from posterior edge of forelimb insertion to anterior edge of hindlimb insertion);

**BW** body width (the widest distance of body);

**IND** internarial distance (distance between nares);

**IOD** interorbital distance (shortest distance between left and right supraciliary scale rows).

Scale counts were taken as follows:

**SupL** Supralabials (counted from the first labial scale to corner of mouth);

**IL** infralabials (counted from the first labial scale to posterior corner of mouth);

**N** nasal scales surrounding nare;

**IN** postrostrals or internasals;

**PM** postmentals;

**GST** granular scales surrounding dorsal tubercles;

**V** ventral scales in longitudianal rows at midbody counted between ventrolateral folds;

**FP** femoral pores;

**PP** precloacal pores;

**PAT** postcloacal tubercles;

**DTR** dorsal tubercle rows (counted transversely across the center of the dorsum from one ventrolateral fold to the other);

**EFS** enlarged femoral scales (number of enlarged femoral scales beneath each thigh);

**LD4** number of subdigital lamellae on fourth finger;

**LT4** number of subdigital lamellae on fourth toe.

### ﻿Statistical analyses

All statistical analyses were conducted using R Core Team (2024). The MFA was applied in this study using the R package FactorMineR (Le et al. 2008) and visualized it using the Factoextra package (Kassambara and Mundt 2017). A concatenated dataset comprised of 12 morphometric (SVL, HL, HW, HH, SE, Eye Ear, ED, ForeaL, TrunkL, IND, IOD) and 10 meristic (SupL (left), Infra (left), V, FP (left/right), PP, TubR, EFS (left/right), NST IV) characters was used as an input for FactorMineR. Other morphological characteristics were not incorporated due to limited available data or incomplete sampling (regenerate tail). To remove the effects of allometry, morphometric data were normalized to adjust raw of morphometrics through the allom function in R package GroupStruct (available at https://github.com/chankinonn/GroupStruct). Accordingly, the allometric formula is X_adj_ = log_10_ (X) – β[log_10_ (SVL) – log_10_ (SVL_mean_)], where X_adj_ = adjusted value; X = measured value; β = unstandardized regression coefficient for each population and SVL_mean_ = overall average SVL of two populations (Thorpe 1975, 1983; Turan 1999; Lleonart et al. 2000; Grismer et al. 2021; Chan and Grismer 2022).

A permutation–based Multivariate Analysis of Variance (perMANOVA) from pairwiseAdonis package in R (available at https://github.com/pmartinezarbizu/pairwiseAdonis/tree/master/pairwiseAdonis) was used to determine if the centroid locations and group clusters of each species were statistically different from each other based on the MFA load scores of dimensions 1–5 (Anderson 2017; Oksanen et al. 2020). A Euclidean (dis) similarity matrix was calculated using 50,000 permutations (Grismer et al. 2024). A pairwise *post hoc* test was also applied to estimate the differences between studied species pairs. A p < 0.05 is considered significant difference between the taxa.

## ﻿Results

### ﻿Phylogenetic analyses

**Figure 2.**
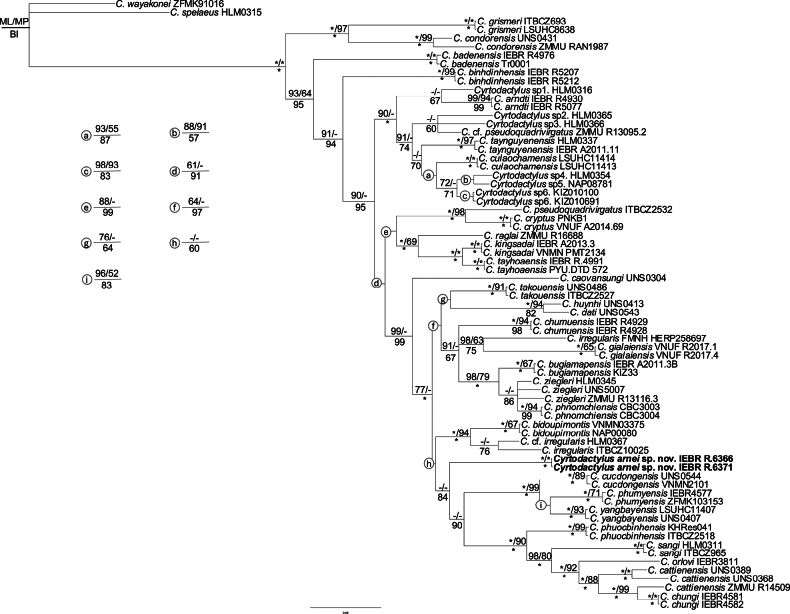
Bayesian phylogram based on the combined matrix of COI and ND2 (+tRNA) genes. Number above and below branches are ML/MP bootstrap and ultrafast bootstrap values and Bayesian posterior probabilities (≥ 50%), respectively.

The matrix of molecular data contained 2,067 aligned characters (COI: 657 and ND2: 1,410), of which 956 were constant, 197 variable and parsimony-uninformative, and 914 parsimony informative. The MP analysis produced a single most parsimonious tree (tree length = 5,435; consistency index = 0.35, retention index = 0.52). Tree topologies from three analyses, ML, MP, and BI based on combined data (COI+ND2) or separate data (COI or ND2) were congruent and similar to those reported in Ngo et al. (2022) and the undescribed species from Hon Tre Island, Khanh Hoa Province was nested within the *Cyrtodactylus irregularis* group without any clear sister taxon (Fig. 2, Suppl. materials 2, 3). Genetically, the new species is divergent from other species within the *Cyrtodactylus irregularis* group by at least ~10.97% (*C. takouensis*) based on a fragment of the mitochondrial COI gene and by at least ~14.39% (*C. bidoupimontis*) based on a fragment of the NADH dehydrogenase subunit 2 (Suppl. material 4: tables S1, S2).

**Morphological analysis.** To date, four known species of *Cyrtodactylus* have been recorded in Khanh Hoa Province, consisting of *C. cucdongensis*, *C. raglai* and *C. yangbayensis* and *C. bidoupimontis* (Ngo and Chan 2010; Schneider et al. 2014; Nguyen et al. 2017, 2021a). Therefore, four representative species in Khanh Hoa Province and *C. takouensis* were included in the MFA analysis.

**Figure 3.**
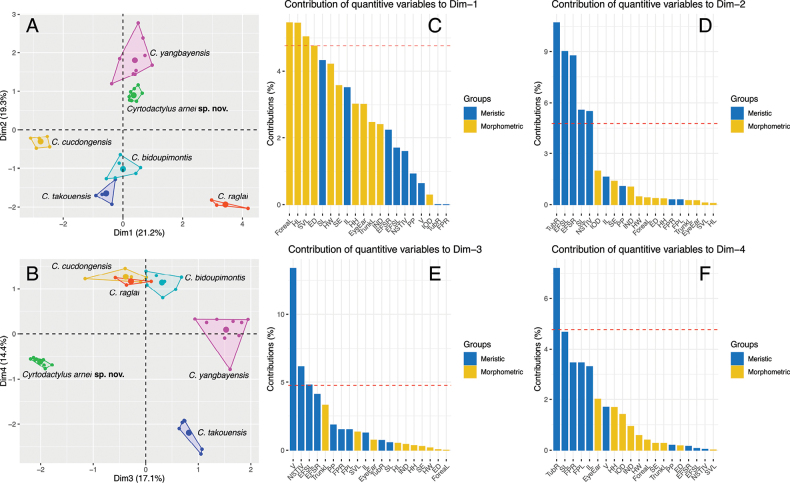
A. MFA of *Cyrtodactylus arnei* sp. nov. and *C. bidoupimontis*, *C. cucdongensis*, *C. takouensis*, and *C. yangbayensis* for Dim 1 and Dim 2 axes; B. MFA of *C. arnei* sp. nov. and *C. bidoupimontis*, *C. cucdongensis*, *C. takouensis*, and *C. yangbayensis* for Dim 3 and Dim 4 axes; C–F. Percent contribution of the quantitative variables to the dimensions 1–4 of the MFA. Dotted red line is the mean percentage if all values were equal.

The MFA analysis revealed that although the new population and *C. bidoupimontis*, *C. yangbayensis* are all overlapped along dimensions 1 and 4 that accounted for 21.2% and 14.4% of the variation in the data set (Fig. 3A, B), respectively. They were distinguished from each other along dimensions 2 and 3 which were attributed to additional 19.3% and 17.1% of the variation, respectively (Fig. 3A, B). Furthermore, the perMANOVA analysis indicated that the new population from Hon Tre Island differs significantly in morphospace from *C. bidoupimontis*, *C. cucdongensis*, *C. raglai*, *C. takouensis* and *C. yangbayensis* (Table 1). Morphometric characters contributed most of the variation along dimension 1 and meristic data contributed to the majority of the variation along dimensions 2, 3 and 4. Morphometric character ForeaL followed by morphometric characters HL, SVL and ED provided major variation along dimension 1 (Fig. 3C), while meristic characters TubR, EFS, SL and NST IV made up most of the variation along dimension 2 (Fig. 3D). Meristic characters V, NST IV and EFS contributed to the majority of the variation along dimension 3 (Fig. 3E), and meristic characters TubR, SL, FP and IL accounted for the majority of the variation along dimension 4 (Fig. 3F).

**Table 1.** Summary statistics from the perMANOVA analysis from the loadings of the MFA comparing *Cyrtodactylus arnei* sp. nov. to *C. bidoupimontis*, *C. cucdongensis*, *C. raglai*, *C. takouensis*, and *C. yangbayensis*.

Species pairs	F.Model	R2	p.value	p.adjusted
*C. arnei* sp. nov. – *C. bidoupimontis*	323.30	0.96	0.10 × 10^–3^	0.14 × 10^–2^
*C. arnei* sp. nov. – *C. cucdongensis*	408.97	0.97	0.58 × 10^–3^	0.52 × 10^–2^
*C. arnei* sp. nov. – *C. raglai*	360.89	0.97	0.41 × 10^–2^	0.021
*C. arnei* sp. nov. – *C. takouensis*	457.16	0.97	0.22 × 10^–3^	0.26 × 10^–2^
*C. arnei* sp. nov. – *C. yangbayensis*.	166.32	0.91	0.60 × 10^–4^	0.90 × 10^–3^

### ﻿Taxonomic account

#### ﻿ Cyrtodactylus arnei sp. nov.

Taxon classificationAnimaliaSquamataGekkonidae

E96A978A-5770-52B8-9690-15A41E844333

https://zoobank.org/2FF533C8-93B7-44B9-AB0A-387EA9A943F1

Figs 4, 5

##### Type material.

***Holotype*.**IEBR R.6365 (Field number KH.2020.7), adult male, collected on 26 September 2020 by C.T. Pham on Hon Tre Island (12°12'58.77"N, 109°15'52.44"E; at an elevation of 74 m asl.), Khanh Hoa Province, Vietnam. ***Paratypes*.** Five adult males: IEBR R.6366 (Field number KH.2020.5), IEBR R.6367 (Field number KH.2020.8), IEBR R.6368 (Field number KH.2020.11), IEBR R.6369 (Field number KH.2020.12), IEBR R.6370 (Field number KH.2020.13); three adult females: IEBR R.6371 (Field number KH.2020.6), IEBR R.6372 (Field number KH.2020.9), IEBR R.6373 (Field number KH.2020.10), the same data as the holotype.

##### Diagnosis.

The new species can be distinguished from other members of the *Cyrtodactylus irregularis* group by a combination of the following characteristics: size medium (SVL 70.9–78.0 mm); dorsal tubercles in 15–17 irregular rows; 34 or 35 ventral scale rows; 12–15 enlarged femoral scales on each side, in continuous series without gap between precloacal and femoral scales; precloacal pores absent in females, 5–7 in males, in a continuous row; femoral pores absent; postcloacal spurs 0–3 on each side; 19–21 lamellae under toe IV; dorsal pattern between limb insertions consisting four narrow light bands with dark edges and a transversal row of dark spots in the middle; subcaudal scales enlarged, forming broad transverse plates.

##### Description of holotype.

Adult male, medium size, snout–vent length (SVL) 74.8 mm. Head wider than body, elongate (HL 21.9 mm, HL/SVL 0.29), wide (HW 13.8 mm, HW/HL 0.63), relatively depressed (HH 7.9 mm, HH/HL 0.36, HH/HW 0.57), distinct from neck; prefrontal and postnasal regions concave; snout elongate (SE/HL 0.45), round anteriorly, longer than orbit diameter (OD/SE 0.5); scales on snout small, round to oval, granular to weakly conical, mostly homogeneous, larger than those on crown, interorbital and occipital regions; orbit of moderate size (OD/HL 0.23), pupils vertical, supraciliaries short, forming conical spines, larger anteriorly; ear opening vertically oval, small in size (ED/HL 0.09), eye to ear distance longer than orbit diameter (Eye Ear/OD 1.23); rostral much wider than deep with a medial suture, bordered by first supralabial on each side, nostrils, two supranasals and one internasal; nostrils oval, each surrounded by supranasal, rostral, first supralabial and three postnasals; two enlarged supranasals separated from one another anteriorly by one internasal; mental triangular, wider than deep; a single pair of greatly enlarged postmentals in broad contact behind mental, bordered by mental anteriorly, first infralabial laterally, and six enlarged chin scale posteriorly; supralabials 12/11; infralabials 10/10; scales of labial area decreasing in size towards jaw.

Body moderately slender, relatively short (TrunkL/SVL 0.37) with the presence of non–denticulate, ventrolateral skin folds; dorsal scales granular; dorsal tubercles round, conical, present on occipital region and back, each surrounded by eight or nine granular scales, in 16 irregular longitudinal rows at midbody; ventral scales larger than dorsal scales, smooth, oval, subimbricate, largest posteriorly, largest on posterior abdomen and in precloacal region; midbody scale rows across belly between ventrolateral folds 36; gular region with homogeneous, smooth, juxtaposed granular scales; 15 or 16 poreless and pitless enlarged femoral scales each thigh, in continuous series with enlarged precloacal scales; precloacal groove absent; precloacal scales arranged in a diamond shape, precloacal pores seven, in a continuous row, pore–bearing scales enlarged; postcloacal spur each bearing three much enlarged conical scales.

**Figure 4.**
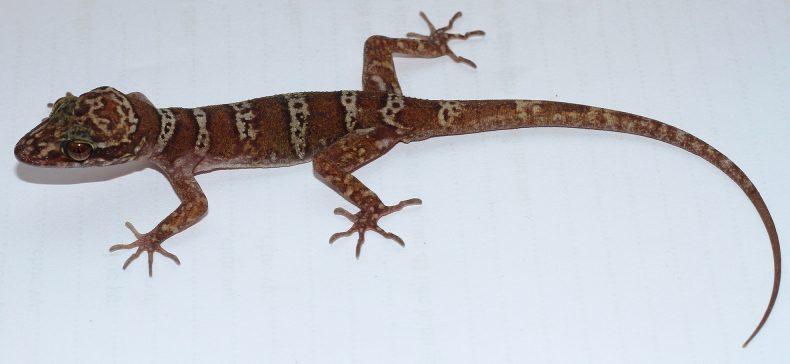
The male holotype of *Cyrtodactylus arnei* sp. nov. (IEBR R.6365, SVL 74.8 mm, TL 107.2 mm) in life. Photograph CTP.

Fore and hind–limbs moderately slender and long (ForeaL/SVL 0.17, CrusL/SVL 0.2); tubercles on dorsum of fore and hind–limbs weakly developed; fingers and toes without distinct webbing; subdigital lamellae: finger IV 17 (with 6 basally broadened lamellae), toe IV 20 (with 7 basally broadened lamellae).

Tail very long (TaL 107.2 mm, TaL/SVL 1.43); subcaudals distinctly transversely enlarged, flat, smooth.

Coloration in life: ground color chocolate brown; dorsal surface of head pale brown with dark reticulated markings, distinct from the lower side by a cream line with dark edge extending from posterior of eye crossing the upper of ear to neck, another line extending from the posterior of labial crossing the ear but interrupted in the neck; eyelids yellowish cream; dorsal pattern consisting of six narrow light bands with dark edges (anterior edges darker than posterior ones) and a transversal row of dark spots in the middle: one on the tail, four between limb insertions and one anterior to the front limbs; original tail chocolate brown, scattered with small darker and cream spots, the same color with front and hind-limbs; ventral side of body cream.

**Figure 5.**
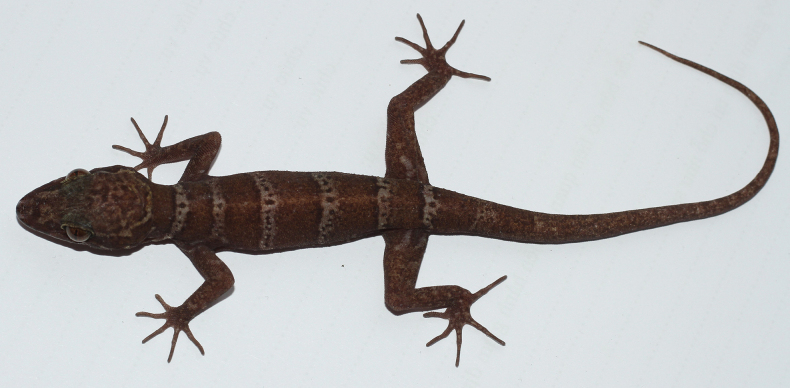
The female paratype of *Cyrtodactylus arnei* sp. nov. (IEBR R.6371, SVL 71.3 mm, TL 103.5 mm) in life. Photos: CTP.

Coloration in preservative: In 70% alcohol, color of this species is slightly faded. The brown turns to grey. Main morphological characters are still clearly visible.

##### Sexual dimorphism and variation.

The females differ from the males in the absence of precloacal pores and hemipenial swellings at the tail base. The number of narrow light band on the tail varies from one to two in each individual. For other morphological characteristics see Table 2.

**Table 2.** Measurements (in mm) and morphological characters of the type series of *Cyrtodactylus arnei* sp. nov. (* = regenerated or broken tail); bilateral meristic characters are given as (left/right).

Characters	IEBR R.6365	IEBR R.6366	IEBR R.6367	IEBR R.6368	IEBR R.6369	IEBR R.6370	IEBR R.6371	IEBR R.6372	IEBR R.6373	Min–max
	Holotype	Paratype	Paratype	Paratype	Paratype	Paratype	Paratype	Paratype	Paratype	
Sex	M	M	M	M	M	M	F	F	F	
SVL	74.8	70.9	73.0	78.0	76.8	75.7	71.3	73.8	71.0	70.9–78.0
TaL	107.2	93.3	104.4	79.8*	100.6	106.8	103.5	106.9	?	93.3–107.2
HL	21.9	20.8	21. 1	22.7	21.7	21.4	21.1	21.0	21.2	20.8–22.7
HW	13.8	13.4	13.8	14.0	13.9	14.0	12.9	12.8	13.1	13.4–14.0
HH	7.9	8.0	8.2	8.0	8.2	8.2	8.0	7.9	7.3	7.3–8.2
OD	4.9	4.9	4.5	5.1	4.7	5.0	5.2	4.7	4.8	4.5–5.2
SE	9.9	9.3	9.3	10.1	9.6	9.2	9.1	9.1	9.6	9.1–10.1
EE	6.1	5.6	5.7	5.7	6.3	5.7	5.4	5.7	5.2	5.2–6.3
NE	7.8	7.0	7.0	7.7	7.2	6.5	6.7	7.2	7.3	6.5–7.8
ED	2.0	1.8	1.8	2.4	2.1	2.1	1.7	1.8	1.7	1.7–2.4
ForeaL	12.4	11.0	12.4	11.9	12.7	12.1	11.7	11.9	11.6	11.0–12.7
CrusL	15.1	14.2	14.5	14.8	15.2	15.0	14.2	14.4	14.8	14.2–15.2
AG	27.8	26.5	26.7	31.3	30.9	29.9	28.9	25.8	26.6	26.5–31.3
BW	13.5	11.3	12.2	12.4	12.0	12.9	12.6	11.8	12.0	11.3–13.5
IND	2.3	2.2	2.3	2.4	2.7	2.2	2.1	2.1	2.1	2.1–2.7
IOD	7.1	5.6	7.2	7.0	6.9	6.4	6.2	5.8	6.2	5.6–7.2
SL	12–11	10–11	11–11	12–11	12–12	11–11	12–11	11–11	11–12	11–12
IL	10–10	10–10	9–9	10–9	10–9	10–10	9–10	10–10	10–9	9–10
N	4–4	4–4	4–4	4–4	3–3	4–4	4–4	4–4	4–4	3–4
IN	1	1	1	1	1	1	1	1	1	1
PM	2	2	2	2	2	2	2	2	2	2
GST	9–9–9	8–9–9	8–9–9	8–9–9	9–9–8	8–9–9	9–9–9	9–9–8	8–9–8	8–9
V	36	35	34	34	34	35	34	35	35	34–35
FP	0	0	0	0	0	0	0	0	0	0
PP	7	6	7	7	5	7	0	0	0	5–7 in males 0 in females
PAT	3–3	2–1	3–2	3–2	3–2	2–3	2–2	1–2	0	0–3
TubR	16	17	16	15	15	16	17	16	16	15–17
EFS	15–16	14	12–13	13–13	14–14	13–15	12–13	12–14	13–13	12–15
NSFIV	17	18	18	17	17	19	17	18	18	17–19
NSTIV	20	21	21	20	19	19	20	21	20	19–21

##### Distribution.

*Cyrtodactylus arnei* sp. nov. is currently known only from the type locality in Hon Tre Island, Khanh Hoa Province (Fig. 1).

##### Etymology.

The new species is named after Dr. Arne Schulze, Executive Director of the Zoological Society for Conservation of Species and Populations (ZGAP) to honor his great commitment and support for herpetological research and conservation in Vietnam, in particular within the scope of the Zoo Species of the Year – The Gecko Conservation Campaign 2024.

##### Ecological notes.

**Figure 6.**
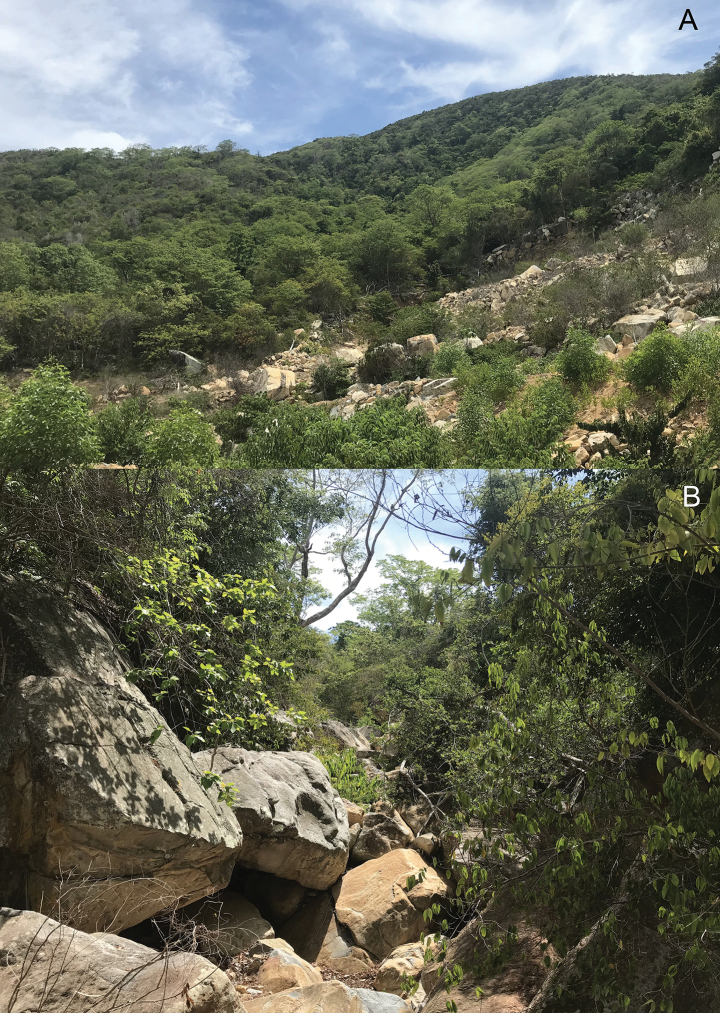
A. Macrohabitat; B. Microhabitat of *Cyrtodactylus arnei* sp. nov. in Hon Tre Island, Khanh Hoa Province, Vietnam. Photo: CTP.

The type series was found from 19:00 to 22:00 hrs on rock boulders and around a small cave along a rocky stream, ~1–2 m above the ground. This is very similar to another granite boulder species in the *C. irregularis* group, *C. raglai* (Nguyen et al. 2021a) to which it is superficially very similar. The surrounding habitat is secondary forest composed of medium and small hardwoods mixed with shrubs (Fig. 6). Other reptile species found at the site were *Calotes versicolor* (Daudin), *Dixonius vietnamensis* Das, *Gekko* sp., and *Eutropis multifasciatus* (Kuhl).

##### Comparisons.

We compared the new species with its 30 congeners from the *Cyrtodactylus irregularis* group based on examination of specimens and data obtained from the literature (Smith 1921; David et al. 2004; Heidrich et al. 2007; Orlov et al. 2007; Nazarov et al. 2008, 2012; Ngo and Bauer 2008; Rösler et al. 2008; Geissler et al. 2009; Ngo and Chan 2010; Ngo 2013; Nguyen et al. 2013, 2021a; Ziegler et al. 2013; Schneider et al. 2014; Luu et al. 2017; Pauwels et al. 2018; Neang et al. 2020; Ngo et al. 2020; Ostrowski et al. 2020, 2021; Do et al. 2021, 2023; Ngo et al. 2023, 2024).(See Table 3).

**Table 3.** Comparisons of the new species with its 30 congeners from the *Cyrtodactylus irregularis* group (measurements in mm, * = regenerated or broken tail, Max = maximum, other abbreviations defined in the text).

No.	Taxa	SVL	TaL	V	EFS	FP	PP(M)	PP (F)	LD4	LT4	Color pattern of dorsum	Enlarged subcaudals
1	*Cyrtodactylus arnei* sp. nov.	70.9–78.0	93.3–107.2	34–35	12–15	absent	5–7	absent	17–19	19–21	banded	present
2	*C. arndti*	73.4–80.9	max. 91.5	26–38	5–11	0–2	6	6	15–20	17–22	banded	present
3	*C. badenensis*	59.3–74.1	58.6–82.4	25–28	absent	absent	absent	absent	?	18–22	banded	present
4	*C. binhdinhensis*	58.5–80.4	max. 84.7	39–42	5–6	10 in males	6–7	5–6	17–18	18–21	banded	present
5	*C. bidoupimontis*	74.0–86.3	75.0–86	38–43	8–10	absent	4–6	absent	15–20	18–23	banded	absent
6	*C. buchardi*	60.0–65.0	46.0–54.0	30	absent	absent	9	?	14	12	blotched	absent
7	*C. bugiamapensis*	58.6–76.8	65.3–83.0	36–46	6–10	absent	7–11	0–7	15–17	17–20	blotched	absent
8	*C. caovansungi*	90.4–94	120	38–44	8	6	9	absent	22	23–25	banded	present
9	*C. cattienensis*	43.5–69	51–64.7	28–42	3–8	absent	6–8	absent	12–16	14–19	banded	absent
10	*C. chungi*	66.6–68.5	62.8*–82.2	30–31	4–6	absent	7	6	15–18	17–20	banded	absent
11	*C. chumuensis*	67.5	51.4*	43–45	4–5	0–2	6–7	?	16–19	17–21	banded	absent
12	*C. cryptus*	62.5–90.8	63.5–88.4	47–50	absent	absent	9–11	absent	18–19	20–23	banded	absent
13	*C. cucdongensis*	55.8–65.9	max. 81.3	41–44	5–9	absent	5–6	4–6	13–18	15–20	banded	absent
14	*C. culaochamensis*	69.8–79.8	89.7–91.2	45–50	absent	absent	7–8	absent	18–19	20–23	banded	absent
15	*C. dati*	max 70.1	max 57.3	42–48	4–7	6–7	5–6	?	?	18–19	blotched	absent
16	*C. gialaiensis*	50.1–62.8	?	38–45	present	absent	9–10	0–8	14–15	15–17	Banded	absent
17	*C. huynhi*	67.2–79.8	61.5–78.6	43–46	3–5	3–8	7–9	0–8 pitted	14–17	17–21	banded	absent
18	*C. irregularis*	72–86	66.0–74	38–45	7–8	absent	5–7	0–6	15–16	18–19	blotched	absent
19	*C. kingsadai*	83–94	max 117	39–46	9–12	3–7	7–9	4–8	19–21	21–25	banded	present
20	*C. orlovi*	61–77.7	max 71.2	36–39	3–8	absent	5–6	absent	15–17	16–19	banded	absent
21	*C. phnomchiensis*	76.1–80.7	56.9–79.1	45–54	0–8	absent	4–5	1–7 pitted	18–20	20–23	banded	Absent
22	*C. phumyensis*	63.6–66.8	?	33–41	5–7	absent	5–7	6 pitted	18–19	18–21	banded	absent
23	*C. phuocbinhensis*	46–60.4	76.1	43–47	5	absent	7	absent	16–21	17–19	striped/blotched	absent
24	*C. pseudoquadrivirgatus*	48.6–83.3	55.7–82.3	41–57	absent	absent	5–9	5–10	15–21	16–25	Varied	absent
25	*C. raglai*	87.5–111.7	113.4–119	36–39	9–10	0	5	0	?	21–22	banded	present
26	*C. sangi*	49.9–56.3	47.9*	37	4	Absent	7	4 (Pitted)	?	?	banded	absent
27	*C. takouensis*	74.7–81.1	77.7–91	39–40	3–5	0–2	3–4	absent	16–17	18–20	banded	present
28	*C. tayhoaensis*	82.9–94.2	max 104.3	37–41	10–11	3–7 males	4–5	absent	20–22	22–24	banded	present
29	*C. taynguyenensis*	60.0–85.0	66.0–94.0	42–49	absent	absent	6	absent	13–18	17–21	blotched	absent
30	*C. yangbayensis*	78.5–92.3	91.3–109.1	39–46	5–16	0–2	6–8	absent	16–19	15–17	banded	Present
31	*C. ziegleri*	84.6–93.0	95.0–107.0	33–39	8–10	0–6	5–8	0–8	16–19	18–21	banded	Absent

Among the 30 species of the *Cyrtodactylus irregularis* group, *Cyrtodactylus arnei* sp. nov. differs:

- from *C. arndti* Ngo, Horman, Le, Pham, Phung, Do, Ostrowski, Nguyen & Zieger by having more enlarged femoral scales (12–15 vs 5–11 in *C. arndti*), the absence of precloacal pores in females (vs 6 in *C. arndti*), and the difference of dorsal color pattern (four narrow light bands with dark edges and a transversal row of dark spots in the middle vs 6 or 7 irregularly shaped bands in *C. arndti*);
- from *C. badenensis* Nguyen, Orlov & Darevsky by having more ventral scale rows (34 or 35 vs 25–28 in *C. badenensis*), the presence of enlarged femoral scales (12–15 vs absent in *C. badenensis*), and the presence of precloacal pores in males (5–7 vs absent in *C. badenensis*);
- from *C. binhdinhensis* Ngo, Do, Do, Pham, Bui, Ho, Nguyen, Ziegler & Le by having fewer ventral scale rows (34 or 35 vs 39–42 in *C. binhdinhensis*), more enlarged femoral scales (12–15 vs 5 or 6 in *C. binhdinhensis*), the absence of femoral pores in males (vs 10 in *C. binhdinhensis*), and the absence of precloacal pores in females (vs 5 or 6 in *C. binhdinhensis*);
- from *C. bidoupimontis* Nazarov, Poyarkov, Orlov, Phung, Nguyen, Hoang & Ziegler by having a longer tail (TL 93.3–107.2 mm, mean ratio TL/SVL 1.40 vs 75–86 mm, ratio TL/SVL 1.05 in *C. bidoupimontis*), fewer ventral scale rows (34 or 35 vs 38–43 in *C. bidoupimontis*), fewer dorsal tubercle rows (15–17 vs 18–24 in *C. bidoupimontis*), more enlarged femoral scales (12–15 vs 8–10 in *C. bidoupimontis*), and the presence of transversely enlarged subcaudal plates (vs absent in *C. bidoupimontis*);
- from *C. buchardi* David, Teynié & Ohler by having a larger size (SVL 70.9–78.0 mm vs 60.0–65.0 mm in *C. buchardi*), more ventral scale rows (34 or 35 vs 30 in *C. buchardi*), the presence of enlarged femoral scales (12–15 vs absent in *C. buchardi*), precloacal pores in males (5–7 vs 9 in *C. buchardi*), more lamellae under finger IV (17–19 vs 14 in *C. buchardi*), more lamellae under toe IV (19–21 vs 12 in *C. buchardi*), and the the difference of dorsal color pattern (banded vs blotched in *C. buchardi*);
- from *C. bugiamapensis* Nazarov, Poyarkov, Orlov, Phung, Nguyen, Hoang & Ziegler by having a longer tail (TL 93.3–107.2 mm, mean ratio TL/SVL 1.40 vs 65.3–83.0 mm, mean ratio TL/SVL 1.08 in *C. bugiamapensis*), fewer ventral scale rows (34 or 35 vs 36–46 in *C. bugiamapensis*), fewer dorsal tubercle rows (15–17 vs 20–24 in *C. bugiamapensis*), more enlarged femoral scales (12–15 vs 6–10 in *C. bugiamapensis*), the difference of dorsal color pattern (banded vs blotched in *C. bugiamapensis*), and the presence of transversely enlarged subcaudal plates (vs absent in *C. bugiamapensis*);
- from *C. caovansungi* Orlov, Nguyen, Roman, Natalia & Nguyen by having a smaller size (70.9–78.0 mm vs 90.4–94 mm in *C. caovansungi*), fewer ventral scale rows (34 or 35 vs 38–44 in *C. caovansungi*), more enlarged femoral scales (12–15 vs 8 in *C. caovansungi*), the absence of femoral pores in males (vs 6 in *C. caovansungi*), fewer precloacal pores in males (5–7 vs 9 in *C. caovansungi*), and fewer lamellae under finger IV (17–19 vs 22 in *C. caovansungi*), under toe IV (19–21 vs 23–25 in *C. caovansungi*);
- from *C. cattienensis* Geissler, Nazarov, Orlov, Böhme, Phung, Nguyen & Ziegler by having a larger size (SVL 70.9–78.0 mm vs 43.5–69.0 mm in *C. cattienensis*), a longer tail (TL 93.3–107.2 mm, mean ratio TL/SVL 1.40 vs 51–64.7 mm, mean ratio TL/SVL 1.07 in *C. cattienensis*), more enlarged femoral scales (12–15 vs 3–8 in *C. cattienensis*), more lamellae under finger IV (17–19 vs 12–16 in *C. cattienensis*), and the presence of transversely enlarged subcaudal plates (vs absent in *C. cattienensis*);
- from *C. chungi* Ostrowski, Le, Ngo, Pham, Phung, Nguyen & Ziegler by having having a larger size (SVL 70.9–78.0 mm vs 66.6–68.5 mm in *C. chungi*), more ventral scale rows (34 or 35 vs 30 or 31 in *C. chungi*), more enlarged femoral scales (12–15 vs 4–6 in *C. chungi*), the absence of precloacal pores in females (vs 6 in *C. chungi*), and the presence of transversely enlarged subcaudal plates (vs absent in *C. chungi*);
- from *C. chumuensis* Ngo, Horman, Le, Pham, Phung, Do, Ostrowski, Nguyen & Zieger by having a larger size (SVL 70.9–78.0 mm vs 67.5 mm in *C. chumuensis*), fewer ventral scale rows (34 or 35 vs 43–45 in *C. chumuensis*), more enlarged femoral scales (12–15 vs 4 or 5 in *C. chumuensis*), and the presence of transversely enlarged subcaudal plates (vs absent in *C. chumuensis*);
- from *C. cryptus* Heidrich, Rösler, Vu, Böhme & Ziegler by having a longer tail (TL 93.3–107.2 mm, mean ratio TL/SVL 1.40 vs 63.5–88.4 mm, mean ratio TL/SVL 1.02 in *C. cryptus*), fewer ventral scale rows (34 or 35 vs 47–50 in *C. cryptus*), fewer dorsal tubercle rows (15–17 vs 19 or 20 in *C. cryptus*), the presence of enlarged femoral scales (12–15 vs absent in *C. cryptus*), fewer precloacal pores in males (5–7 vs 9–11 in *C. cryptus*), and the presence of transversely enlarged subcaudal plates (vs absent in *C. cryptus*);
- from *C. cucdongensis* Schneider, Phung, Le, Nguyen & Ziegler by having a larger size (SVL 70.9–78.0 mm vs 55.8–65.9 mm in *C. cucdongensis*), fewer ventral scale rows (34 or 35 vs 41–44 in *C. cucdongensis*), more enlarged femoral scales (12–15 vs 5–9 in *C. cucdongensis*), the absence of precloacal pores in females (vs 4–6 in *C. cucdongensis*), and the presence of transversely enlarged subcaudal plates (vs absent in *C. cucdongensis*);
- from *C. culaochamensis* Ngo, Grismer, Pham & Wood by having a longer tail (TL 93.3–107.2 mm, mean ratio TL/SVL 1.40 vs 89.7–91.2 mm, mean ratio TL/SVL 1.24 in *C. culaochamensis*), fewer ventral scale rows (34 or 35 vs 45–50 in *C. culaochamensis*), fewer dorsal tubercle rows (15–17 vs 20–22 in *C. culaochamensis*), the presence of enlarged femoral scales (12–15 vs absent in *C. culaochamensis*), and the presence of transversely enlarged subcaudal plates (vs absent in *C. culaochamensis*);
- from *C. dati* Ngo by having a larger size (SVL 70.9–78.0 mm vs max 70.1 in *C. dati*), a longer tail (TL 93.3–107.2 mm, mean ratio TL/SVL 1.40 vs Max 57.3 mm, mean ratio TL/SVL: 1.06), fewer ventral scale rows (34 or 35 vs 42–48 in *C. dati*), fewer dorsal tubercle rows (15–17 vs 20–22 in *C. dati*), more enlarged femoral scales (12–15 vs 4–7 in *C. dati*), the absence of femoral pores in males (vs 6–7 in *C. dati*), the difference in color pattern of dorsum (banded vs small blotched in *C. dati*), and the presence of transversely enlarged subcaudal plates (vs absent in *C. dati*);
- from *C. gialaiensis* Luu, Tran, Nguyen, Le & Ziegler by having a larger size (SVL 70.9–78.0 mm vs 50.1–62.8 mm in *C. gialaiensis*), fewer ventral scale rows (34 or 35 vs 38–45 in *C. gialaiensis*), fewer precloacal pores in males (5–7 vs 9 or 10 in *C. gialaiensis*), the absence of precloacal pores in adult females (vs 8 pitted scales in *C. gialaiensis*), more lamellae under finger IV (17–19 vs 14 or 15 in *C. gialaiensis*) and under toe IV (19–21 vs 15–17 in *C. gialaiensis*), and the presence of transversely enlarged subcaudal plates (vs absent in *C. gialaiensis*);
- from *C. huynhi* Ngo & Bauer by having fewer ventral scale rows (34 or 35 vs 43–46 in *C. huynhi*), more enlarged femoral scales (12–15 vs 3–5 in *C. huynhi*), the absence of femoral pores in males (vs 3–8 in *C. huynhi*), and the presence of transversely enlarged subcaudal plates (vs absent in *C. huynhi*);
- from *C. irregularis* (Smith) by having fewer ventral scale rows (34 or 35 vs 38–45 in *C. irregularis*), more enlarged femoral scales (12–15 vs 7 or 8 in *C. irregularis*), the absence of precloacal pores in females (0 vs 0–6 in *C. irregularis*), the difference in color pattern of dorsum (banded vs blotched in *C. irregularis*), and the presence of transversely enlarged subcaudal plates (vs absent in *C. irregularis*);
- from *C. kingsadai* Ziegler, Phung, Le & Nguyen by having a smaller size (SVL 70.9–78.0 mm vs 83–94 mm in *C. kingsadai*), fewer ventral scale rows (34 or 35 vs 39–46 in *C. kingsadai*), the absence of femoral pores in males (vs 3–7 in *C. kingsadai*), and the absence of precloacal pores in females (vs 4–8 in *C. kingsadai*);
- from *C. orlovi* Do, Phung, Ngo, Le, Ziegler, Pham & Nguyen by having fewer ventral scale rows (34 or 35 vs 36–39 in *C. orlovi*), more enlarged femoral scales (12–15 vs 3–8 in *C. orlovi*), and the presence of transversely enlarged subcaudal plates (vs absent in *C. orlovi*);
- from *C. phnomchiensis* Neang, Henson & Stuart by having a longer tail (TL 93.3–107.2 mm, mean ratio TL/SVL 1.40 vs 56.9–79.1 mm, mean ratio TL/SVL 0.88 in *C. phnomchiensis*), fewer ventral scale rows (34 or 35 vs 45–54 in *C. phnomchiensis*), more enlarged femoral scales (12–15 vs 0–8 in *C. phnomchiensis*), the absence of precloacal pores in females (vs 1–7 pitted scales in *C. phnomchiensis*), and the presence of transversely enlarged subcaudal plates (vs absent in *C. phnomchiensis*);
- from *C. phumyensis* Ostrowski, Le, Ngo, Pham, Phung, Nguyen & Ziegler by having a larger size (SVL 70.9–78.0 mm vs 63.6–66.8 mm in *C. phumyensis*), fewer dorsal tubercle rows (15–17 vs 18–19 in *C. phumyensis*), more enlarged femoral scales (12–15 vs 5–7 in *C. phumyensis*), the absence of precloacal pores in females (vs 6 pitted scales in *C. phumyensis*), and the presence of transversely enlarged subcaudal plates (vs absent in *C. phumyensis*);
- from *C. phuocbinhensis* Nguyen, Le, Tran, Orlov, Lathrop, Macculloch, Le, Jin, Nguyen, Nguyen, Hoang, Che, Murphy & Zhang by having a larger size (SVL 70.9–78.0 mm vs 46.0–60.4 mm in *C. phuocbinhensis*), fewer ventral scale rows (34 or 35 vs 43–47 in *C. phuocbinhensis*), more enlarged femoral scales (12–15 vs 5 in *C. phuocbinhensis*), the difference of dorsal color pattern (banded vs blotched/striped in *C. phuocbinhensis*), and the presence of transversely enlarged subcaudal plates (vs absent in *C. phuocbinhensis*);
- from *C. pseudoquadrivirgatus* Rösler, Vu, Nguyen, Ngo & Ziegler by having fewer ventral scale rows (34 or 35 vs 41–57 in *C. pseudoquadrivirgatus*), the presence of enlarged femoral scales (12–15 vs absent in *C. pseudoquadrivirgatus*), the absence of precloacal pores in females (vs 5–10 in *C. pseudoquadrivirgatus*), and the presence of transversely enlarged subcaudal plates (vs absent in *C. pseudoquadrivirgatus*);
- from *C. raglai* Nguyen, Duong, Grismer & Poyarkov by having a smaller size (70.9–78.0 mm vs 87.5–111.7 mm in *C. raglai*), fewer ventral scale rows (34 or 35 vs 36–39 in *C. raglai*), and more enlarged femoral scales (12–15 vs 9–10 in *C. raglai*);
- from *C. sangi* Pauwels, Nazarov, Bobrov & Poyarkov by having a larger size (SVL 70.9–78.0 mm vs 49.9–56.3 mm in *C. sangi*), fewer ventral scale rows (34 or 35 vs 37 in *C. sangi*), more enlarged femoral scales (12–15 vs 4 in *C. sangi*), the absence of precloacal pores in females (vs 4 pitted scales in *C. sangi*), and the presence of transversely enlarged subcaudal plates (vs absent in *C. sangi*);
- from *C. takouensis* Ngo & Bauer by having fewer ventral scale rows (34 or 35 vs 39–40 in *C. takouensis*), more dorsal tubercle rows (15–17 vs 9–10 in *C. takouensis*), more enlarged femoral scales (12–15 vs 3–5 in *C. takouensis*), and more precloacal pores in males (5–7 vs 3–4 in *C. takouensis*);
- from *C. tayhoaensis* by having a smaller size (SVL 70.9–78.0 mm vs 82.9–94.2 mm in *C. tayhoaensis*), fewer ventral scale rows (34 or 35 vs 37–41 in *C. tayhoaensis*), fewer dorsal tubercle rows (15–17 vs 20–22 in *C. tayhoaensis*), the absence of femoral pores in males (vs 3–7 in *C. tayhoaensis*), and fewer lamellae under finger IV (17–19 vs 20–22 in *C. tayhoaensis*), and under toe IV (19–21 vs 22–24 in *C. tayhoaensis*);
- from *C. taynguyenensis* Nguyen, Le, Tran, Orlov, Lathrop, Macculloch, Le, Jin, Nguyen, Nguyen, Hoang, Che, Murphy & Zhang by having fewer ventral scale rows (34 or 35 vs 42–49 in *C. taynguyenensis*), the presence of enlarged femoral scales (12–15 vs absent in *C. taynguyenensis*), the difference in color pattern of dorsum (banded vs blotched in *C. taynguyenensis*), and the presence of transversely enlarged subcaudal plates (vs absent in *C. taynguyenensis*);
- from *C. yangbayensis* Ngo & Chan by having fewer ventral scale rows (34 or 35 vs 39–46 in *C. yangbayensis*), fewer dorsal tubercle rows (15–17 vs 20–23 in *C. yangbayensis*), the absence of femoral pores in males (vs 0–2 in *C. yangbayensis*), and more lamellae under under toe IV (19–21 vs 15–17 in *C. yangbayensis*);
- from *C. ziegleri* Nazarov, Orlov, Nguyen & Ho by having a smaller size (SVL 70.9–78.0 mm vs 84.6–93.0 mm in *C. ziegleri*), more enlarged femoral scales (12–15 vs 8–10 in *C. ziegleri*), the absence of femoral pores in males (vs 0–6 in *C. ziegleri*), the absence precloacal pores in females (vs 0–8 in *C. ziegleri*), and the presence of transversely enlarged subcaudal plates (vs absent in *C. ziegleri*).

## ﻿Discussion

*Cyrtodactylus arnei* is the fifth species of *Cyrtodactylus* recorded in Khanh Hoa Province. However, *Cyrtodactylus arnei* differs significantly from the four previously known species, *C. bidoupimontis*, *C. cucdongensis*, *C. raglai*, and *C. yangbayensis* by a number of morphological characteristics. Phylogenetically, it is also not closely related to those species (Fig. 2). It bears an uncorrected pairwise sequence divergence of approximately 11.81%, 12.57%, 12.30% and 11.71–11.96% from *C. bidoupimontis*, *C. cucdongensis*, *C. raglai*, and *C. yangbayensis*, respectively (Suppl. material 1). Furthermore, *Cyrtodactylus arnei* is only known from Hon Tre Island in Khanh Hoa Province, where no representative of the genus *Cyrtodactylus* has been reported so far. The new species is the 31^st^ species of the *C. irregularis* group and the 55^th^ species of *Cyrtodactylus* known from Vietnam (Uetz et al. 2025).

Khanh Hoa Province is located in South–central of Vietnam. It has a long coastline and nearly 200 islands. Hon Tre Island is a small island in Nha Trang Bay, Khanh Hoa Province and it is different from another island also named “Hon Tre” located in the Gulf of Thailand in Vietnam. It is located ca 2.1 km from the coast and 5.67 km long from north to south and 12 km wide from west to east (Nguyen et al. 2022b). The island has recently become a popular tourist destination, and rapid tourism development will likely negatively impact the surrounding ecosystems. Therefore, there is an urgent need for further studies to provide a better understanding of the new species’ population status, distribution range, and anthropogenic threats.

## Supplementary Material

XML Treatment for Cyrtodactylus arnei

## ﻿Additional information

### Use of AI

No use of AI was reported.

### Author ORCIDs

Quyen Hanh Do https://orcid.org/0000-0002-9437-4673

Truong Quang Nguyen https://orcid.org/0000-0002-6601-0880

Minh Duc Le https://orcid.org/0000-0002-2953-2815

Thomas Ziegler https://orcid.org/0000-0002-4797-609X

Dang Trong Do https://orcid.org/0000-0003-3048-1434

Cuong The Pham https://orcid.org/0000-0001-5158-4526

### Data availability

All of the data that support the findings of this study are available in the main text or Supplementary Information.

## Supplementary materials

Supplementary material 1 Species of *Cyrtodactylus* used in the phylogenetic analysis including localities and GenBank accession numbers of the mitochondrial COI and ND2 fragment genes Data type docx https://binary.pensoft.net/file/1423504This dataset is made available under the Open Database License (http://opendatacommons.org/licenses/odbl/1.0/). The Open Database License (ODbL) is a license agreement intended to allow users to freely share, modify, and use this Dataset while maintaining this same freedom for others, provided that the original source and author(s) are credited.Quyen Hanh Do, Hanh Thi Ngo, Truong Quang Nguyen, Minh Duc Le, Thomas Ziegler, Dang Trong Do, Cuong The Pham

Supplementary material 2 Bayesian phylogram based on the matrix of COI. Number above and below branches are ultrafast bootstrap values and Bayesian posterior probabilities (≥ 50%), respectively Data type pdf https://binary.pensoft.net/file/1423505This dataset is made available under the Open Database License (http://opendatacommons.org/licenses/odbl/1.0/). The Open Database License (ODbL) is a license agreement intended to allow users to freely share, modify, and use this Dataset while maintaining this same freedom for others, provided that the original source and author(s) are credited.Quyen Hanh Do, Hanh Thi Ngo, Truong Quang Nguyen, Minh Duc Le, Thomas Ziegler, Dang Trong Do, Cuong The Pham

Supplementary material 3 Bayesian phylogram based on the matrix of ND2. Number above and below branches are ultrafast bootstrap values and Bayesian posterior probabilities (≥ 50%), respectively Data type pdf https://binary.pensoft.net/file/1423506This dataset is made available under the Open Database License (http://opendatacommons.org/licenses/odbl/1.0/). The Open Database License (ODbL) is a license agreement intended to allow users to freely share, modify, and use this Dataset while maintaining this same freedom for others, provided that the original source and author(s) are credited.Quyen Hanh Do, Hanh Thi Ngo, Truong Quang Nguyen, Minh Duc Le, Thomas Ziegler, Dang Trong Do, Cuong The Pham

Supplementary material 4 Pair–wise genetic distance between samples used in this study based on COI and ND2 Data type xlsx https://binary.pensoft.net/file/1423507This dataset is made available under the Open Database License (http://opendatacommons.org/licenses/odbl/1.0/). The Open Database License (ODbL) is a license agreement intended to allow users to freely share, modify, and use this Dataset while maintaining this same freedom for others, provided that the original source and author(s) are credited.Quyen Hanh Do, Hanh Thi Ngo, Truong Quang Nguyen, Minh Duc Le, Thomas Ziegler, Dang Trong Do, Cuong The Pham
