# Supplementary material for: ﻿A new species of Cyrtodactylus (Squamata, Gekkonidae) from Hon Tre Island in Khanh Hoa Province, Vietnam
Source: Zookeys. 2025 Sep 24;1253:195–218. doi: 10.3897/zookeys.1253.149459 (PMC12489490; doi:10.3897/zookeys.1253.149459)
Supplement: Supplementary material 1 — Species of Cyrtodactylus used in the phylogenetic analysis including localities and GenBank accession numbers of the mitochondrial COI and ND2 fragment genes [file zookeys-1253-195_article-149459__-s001.docx]

**Supplementary 1**. Species of *Cyrtodactylus* used in the phylogenetic analysis including localities and GenBank accession numbers of the mitochondrial COI and ND2 fragment genes

| **Species** | **Locality** | **Museum number/Field number** | **ND2** | **COI** |
| --- | --- | --- | --- | --- |
| *C. spelaeus* | Laos: Vientiane, Kasi | HLM0315 | MW713962 | - |
| *C. wayakonei* | Laos: Luang Nam Tha Prov., Vieng Phoukha District, Ban Nam Eng, Kao Rao Cave | ZFMK91016 | MT953498 | KJ817438 |
| *Cyrtodactylus arnei* **sp. nov.** | Vietnam: Khanh Hoa Prov., Hon Tre Island | IEBR R.6366 | XXXXXX | XXXXXX |
| *Cyrtodactylus arnei* **sp. nov.** | Vietnam: Khanh Hoa Prov., Hon Tre Island | IEBR R.6371 | XXXXXX | XXXXXX |
| *C. arndti* | Vietnam: Binh Dinh Prov., Van Canh District | IEBR-R.5077 | - | OQ152316 |
| *C. arndti* | Vietnam: Binh Dinh Prov., Van Canh District | IEBR-R.4930 | - | OQ152317 |
| *C. badenensis* | Vietnam: Tay Ninh Prov., Ba Den Mt | IEBR4976 | - | ON145836 |
| *C. badenensis* | Vietnam: Tay Ninh Prov., Ba Den Mt | TR0001 | MT953468 | ON145835 |
| *C. bidoupimontis* | Vietnam: Khanh Hoa Prov., Nha Trang | VNMN03375 | MT953470 | - |
| *C. bidoupimontis* | Vietnam: Lam Dong Prov., Bidoup-Nui Ba NP | ZMMU NAP00080 | - | KC016074 |
| *C. binhdinhensis* | Vietnam: Binh Dinh Prov., Phu Cat District | IEBR R.5207 | - | PP441921 |
| *C. binhdinhensis* | Vietnam: Binh Dinh Prov., Phu Cat District | IEBR R.5212 | - | PP441922 |
| *C. bugiamapensis* | Vietnam: Binh Phuoc Prov., Bu Gia Map NP | IEBR A2011.3B | MT953473 | ON145810 |
| *C. bugiamapensis* | Vietnam: Binh Phuoc Prov., Bu Gia Map NP | KIZ00033 | - | KY862173 |
| *C. caovansungi* | Vietnam: Ninh Thuan Prov., Nui Chua NP | UNS0304 | MF169954 | - |
| *C. cattienensis* | Vietnam: Dong Nai Prov., Ma Da SFE | UNS0389 | MF169956 | - |
| *C. cattienensis* | Vietnam: Ba Ria - Vung Tau Prov., Binh Chau - Phuoc Buu NR | ZMMU_R14509 | - | MG791892 |
| *C. cattienensis* | Vietnam: Dong Nai Prov., Ma Da SFE | UNS0368 | MF169955 | - |
| *C. chumuensis* | Vietnam: Dak Lak Prov., M’Drak District | IEBR-R.4928 | - | OQ152319 |
| *C. chumuensis* | Vietnam: Dak Lak Prov., M’Drak District | IEBR-R.4929 | - | OQ152320 |
| *C. chungi* | Vietnam: Binh Thuan Prov., Ta Kou NR | IEBR 4581 | - | MT576019 |
| *C. chungi* | Vietnam: Binh Thuan Prov., Ta Kou NR | IEBR 4582 | - | MT576020 |
| *C. condorensis* | Cambodia: Koh Tang Island | ZMMU RAN 1987 | - | HM888464 |
| *C. condorensis* | Vietnam: Ba Ria - Vung Tau Prov., Con Dao Island | UNS 0431 | MF169958 | MF169910 |
| *C. cryptus* | Vietnam: Quang Binh Prov., Phong Nha – Ke Bang NP | PNKB 1 | - | KF169969 |
| *C. cryptus* | Laos: Khammouane Prov., Hin Nam No NPA | VNUF A2014.69 | MT953476 | KX064038 |
| *C. cucdongensis* | Vietnam: Khanh Hoa Prov., Hon Heo Mountain | UNS0544 | MF169959 | - |
| *C. cucdongensis* | Vietnam: Khanh Hoa Prov., Cuc Dong Cape | VNMN2101 | - | MG791883 |
| *C. culaochamensis* | Vietnam: Quang Nam Prov., Cu Lao Cham Island | LSUHC11413 | KT013198 | - |
| *C. culaochamensis* | Vietnam: Quang Nam Prov., Cu Lao Cham Island | LSUHC11414 | KT013199 | - |
| *C. dati* | Vietnam: Binh Phuoc Prov., Bu Dop | UNS0543 | MF169960 | - |
| *C. gialaiensis* | Vietnam: Gia Lai Prov., Chu Se District | VNUF R.2017.1 | MT953479 | MG460299 |
| *C. gialaiensis* | Vietnam: Gia Lai Prov., Chu Se District | VNUF R.2017.4 | - | MG460300 |
| *C. grismeri* | Vietnam: An Giang Prov., Tuc Dup Mt. | ITBCZ 693 | - | KF929516 |
| *C. grismeri* | Vietnam: An Giang Prov., Tuc Dup Mt. | LSUHC8638 | JX440538 | ON145806 |
| *C. huynhi* | Vietnam: Dong Nai Prov., Chua Chan Mt. | UNS0413 | MF169963 | - |
| *C. irregularis* | Laos: Champasal Prov., Pakxong District. | FMNH HERP258697 | JX041341 | - |
| *C.* cf*. irregularis* | Vietnam: Lam Dong Prov., Loc Bac | HLM367 | MW713952 | - |
| *C. kingsadai* | Vietnam: Dak Nong Prov. | VNMN PMT2134 | - | ON145834 |
| *C. kingsadai* | Vietnam: Phu Yen Prov., Tuy Hoa District, Dai Lanh Cape | IEBR A2013.3 | MT953483 | KF188432 |
| *C. orlovi* | Vietnam: Ninh Thuan Prov., Thuan Nam District | IEBR 3811 | - | MZ440851 |
| *C. phnomchiensis* | Cambodia: Kampong Thom Prov., Sandan District | CBC 3003 | - | MT066405 |
| *C. phnomchiensis* | Cambodia: Kampong Thom Prov., Sandan District | CBC 3004 | - | MT066406 |
| *C. phumyensis* | Vietnam: Binh Dinh Prov., Phu My District | ZFMK:103153 | MW792065 | MT210158 |
| *C. phumyensis* | Vietnam: Binh Dinh Prov., Phu My District | IEBR:4577 | - | MT210161 |
| *C. phuocbinhensis* | Vietnam: Ninh Thuan Prov., Phuoc Binh District | ITBCZ 1518 | - | KF169953 |
| *C. phuocbinhensis* | Vietnam: Khanh Hoa Prov., O Kha Valley | KHReS041 | MT953488 | ON145822 |
| *C.* cf. *pseudoquadrivirgatus* | Vietnam | ZMMU R13095.2 | - | KP199949 |
| *C. pseudoquadrivirgatus* | Vietnam: Da Nang Prov., Ba Na | ITBCZ 2532 | - | KF169962 |
| *C. raglai* | Vietnam: Khanh Hoa Prov., Khanh Vinh District | ZMMU R16688 | MW675652 | MW675653 |
| *C. sangi* | Vietnam: Khanh Hoa Prov., Cam Ranh | HLM0311 | MW713956 | - |
| *C. sangi* | Vietnam: Ninh Thuan Prov., Nui Chua NP | ITBCZ965 | - | KF169952 |
| *C. takouensis* | Vietnam: Binh Thuan Prov., Ta Kou NR | ITBCZ 2527 | - | KF929533 |
| *C. takouensis* | Vietnam: Binh Thuan Prov., Ta Kou NR | UNS0486 | MF169978 | - |
| *C. tayhoaensis* | Vietnam: Phu Yen Prov., Tay Hoa District | IEBR R.4991 | - | OQ603084 |
| *C. tayhoaensis* | Vietnam: Phu Yen Prov., Tay Hoa District | PYU.DTD 572 | - | OQ603085 |
| *C. taynguyenensis* | Vietnam: Kon Tum Prov., Mang Canh | HLM0337 | MW713953 | - |
| *C. taynguyenensis* | Vietnam: Gia Lai Prov., Kon Ka Kinh | IEBR A2011.11 | - | KY862145 |
| *C. yangbayensis* | Vietnam: Khanh Hoa Prov., Hon Ba NR | UNS0407 | MF169980 | - |
| *C. yangbayensis* | Vietnam: Khanh Hoa Prov., Hon Ba NR | LSUHC11407 | KT013202 | - |
| *C. ziegleri* | Vietnam: Dak Lak Prov., Chu Yang Sin NP | UNS 5007 | - | KF169945 |
| *C. ziegleri* | Vietnam: Dak Lak Prov., Chu Yang Sin NP | ZMMU R-13116-3 | - | HQ967210 |
| *C. ziegleri* | Vietnam: Dak Lak Prov., Yok Don | HLM0345 | MW713968 | - |
| *Cyrtodactylus* sp1 | Vietnam: Gia Lai Prov., Kon Ka Kinh | HLM0316 | MW713951 | - |
| *Cyrtodactylus* sp2 | Vietnam: Gia Lai Prov., Kon Ka Kinh | HLM0365 | MW713950 | - |
| *Cyrtodactylus* sp3 | Vietnam: Kon Tum Prov., Chu Mom Ray NP. | HLM0366 | MW713954 | - |
| *Cyrtodactylus* sp4 | Vietnam: Kon Tum Prov., Kon Plong | HLM0354 | MW713955 | - |
| *Cyrtodactylus* sp5 | Vietnam: Quang Nam Pro., Song Thanh | NAP08781 | MW713949 | - |
| *Cyrtodactylus* sp6 | Vietnam: Thua Thien Hue, Phong Dien District | KIZ010100 | - | KY862131 |
| *Cyrtodactylus* sp6 | Vietnam: Thua Thien Hue, Phong Dien District | KIZ010691 | - | KY862132 |

Notes. Prov. = Province, NP = National Park, NR = Nature Reserve, Mt. = mountain
