# Supplementary figures and images for: ﻿A new species of Cyrtodactylus (Squamata, Gekkonidae) from Hon Tre Island in Khanh Hoa Province, Vietnam
Source: Zookeys. 2025 Sep 24;1253:195–218. doi: 10.3897/zookeys.1253.149459 (PMC12489490; doi:10.3897/zookeys.1253.149459)

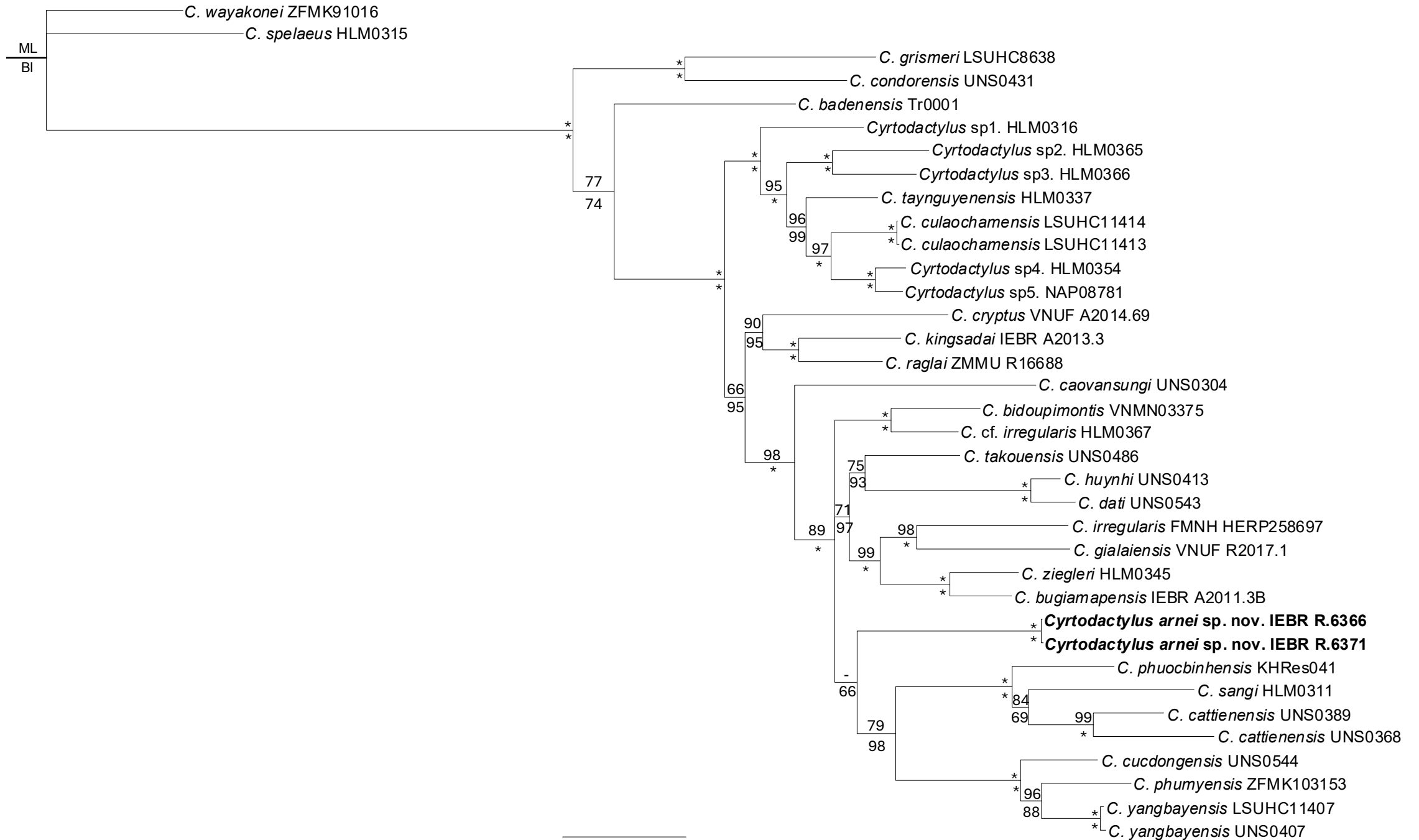

0.1 substitutions/site

Supplement: Supplementary material 2 — Bayesian phylogram based on the matrix of COI. Number above and below branches are ultrafast bootstrap values and Bayesian posterior probabilities (≥ 50%), respectively [file zookeys-1253-195_article-149459__-s002.pdf]

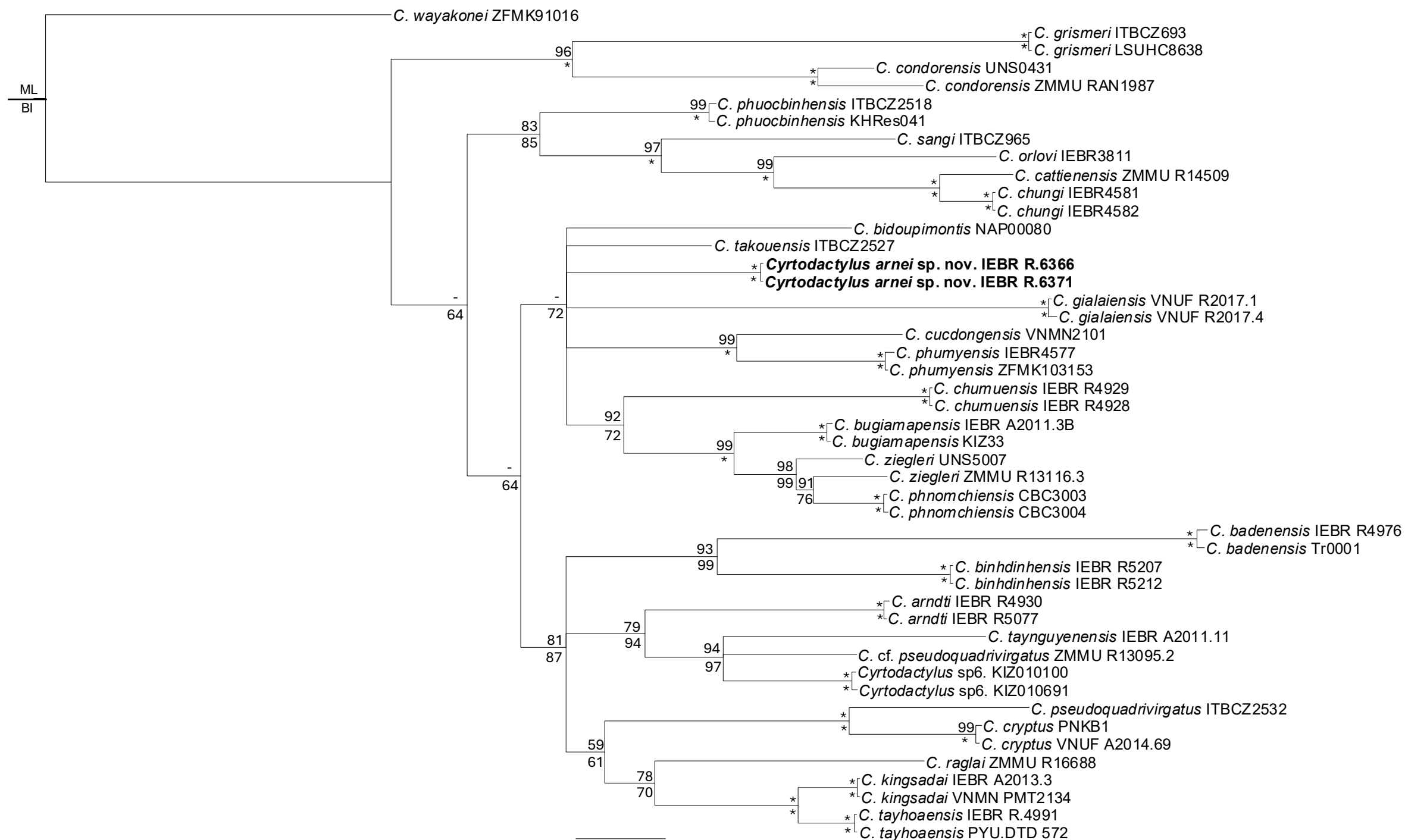

0.04 substitutions/site

Supplement: Supplementary material 3 — Bayesian phylogram based on the matrix of ND2. Number above and below branches are ultrafast bootstrap values and Bayesian posterior probabilities (≥ 50%), respectively [file zookeys-1253-195_article-149459__-s003.pdf]
